# Improving the Detection Performance of Cardiovascular Diseases from Heart Sound Signals with a New Deep Learning-Based Approach

**DOI:** 10.3390/diagnostics15182379

**Published:** 2025-09-18

**Authors:** Ozgen Safak, Mehmet Tolga Hekim, Tolga Cakmak, Fatih Demir, Kursat Demir

**Affiliations:** 1 Clinics of Cardiology, Balıkesir University, Balıkesir 10185, Turkey; ozgen_safak@yahoo.com (O.S.); tolgahekim@yahoo.com (M.T.H.); 2 Clinics of Cardiology, Balıkesir Atatürk City Hospital, Balıkesir 10050, Turkey; tolgacakmak85@gmail.com; 3 Software Department, Engineering Faculty, Firat University, Elazig 23119, Turkey; 4 Mechatronics Department, Technology Faculty, Firat University, Elazig 23119, Turkey; kursatdemir62@gmail.com

**Keywords:** cardiovascular diseases, PCG signals, RAMM model, NRBMI algorithm

## Abstract

**Background/Objectives**: Cardiovascular diseases are among the leading causes of death worldwide. Early diagnosis of these conditions minimizes the risk of future death. Listening to heart sounds with a stethoscope is one of the easiest and fastest methods for diagnosing heart conditions. While heart sounds are a quick and easy diagnostic method, they require significant expert interpretation. Recently, artificial intelligence models trained based on these expert interpretations have become popular in the development of decision support systems. **Methods**: The proposed approach uses the popular 2016 PhysioNet/CinC Challenge dataset for PCG signals. Spectrogram image transformation was then performed to increase the representativeness of these signals. A deep learning-based model that allows for the simultaneous training of residual and attention blocks and the MLP-mixer model was used for feature extraction. A new algorithm combining the strengths of NCA and ReliefF algorithms was proposed to select the strongest features in the feature set. The SVM algorithm was used for classification. **Results**: With this proposed approach, over 98% success was achieved in all accuracy, sensitivity, specificity, precision, and F1-score metrics. **Conclusions**: As a result, an artificial intelligence-based decision support system that detects cardiovascular diseases with high accuracy is presented.

## 1. Introduction

According to WHO (2023), more than 70% of deaths worldwide are caused by noncommunicable diseases, such as cardiovascular disease. Over three-quarters of these deaths occur in low- and middle-income countries [1]. Heart auscultation is a technique where physicians use stethoscopes to record and examine heart sounds for an initial assessment of the heart and lungs. However, its accuracy depends heavily on the clinician’s expertise, making the results subjective [2]. This limitation is more evident in underdeveloped areas with limited medical resources, where highly qualified physicians are scarce. It creates a serious obstacle to diagnosing cardiovascular diseases.

Heart sound signals, produced when heart valves open and close during the cardiac cycle, carry rich physiological information [3]. They are crucial indicators for diagnosing cardiovascular disorders, especially valve diseases. Low frequencies produced by the heart vibrations make up the majority of heart sounds [4]. Murmurs, on the other hand, are abnormal sounds produced during heart function [5], providing vital information for the diagnosis of heart diseases. As a result, heart sound signals serve as a crucial diagnostic tool for cardiovascular conditions, such as arrhythmias, ventricular septal defects, and valve disorders [6]. Automated detection of cardiovascular disease can be achieved using PCG signal data and related technology. This approach improves diagnostic precision and is especially useful in areas with limited medical resources. It also has significant real-world applications [7]. Heart sound signals are nonlinear and time-varying due to muscle tremors, respiratory noises, and lung sounds. Environmental noise and device interference further complicate feature extraction and analysis. Researchers have been looking into more efficient signal-processing techniques to get around these obstacles [8].

Researchers first used conventional techniques such as wavelet transforms and filters for feature learning. However, these methods have limitations in detecting complex heart sound patterns. Francesco Renna et al. [5] used an end-to-end learning deep convolutional neural network (CNN). They did this by taking advantage of the Butterworth filter’s smooth transition characteristics. The average sensitivity of this approach was 93.9%. A different approach was taken by Omer Deperlioglu et al. [9], who used a computerized elliptical filter for reducing heart noise. The signal-to-noise ratio was further improved by effectively removing the noise variables in the heart sounds by adjusting the filter components, which made use of the elliptical filter’s steep transition characteristics and the most suitable working conditions between the pass and stop bands. In order to extract heart sound features, Na Mei et al. [10] thoroughly investigated the time–frequency domain properties of heart sound signals using wavelet scattering transform technology. They then used a robust classifier, support vector machines (SVMs), to categorize the retrieved features as either normal or aberrant. This technique offers robust support for the early identification of heart diseases in addition to increasing the classification accuracy of heart sound signals. Time-domain feature extraction was thoroughly studied by Akram et al. [11]. They retrieved hand-crafted features that thoroughly boost the performance of heart sound data in several aspects. To further enhance the feature collection, they additionally integrated these characteristics with discrete wavelet transform (DWT) coefficients. New concepts for heart sound signal analysis were generated by successfully identifying key heart sound signals in SVM algorithms by concatenating time-domain data with DWT coefficient parameters.

To create temporal and time–frequency features, Nia et al. [6] presented a novel automated PCG data categorization technique that concatenates sequential forward feature selection and particle swarm optimization. Through the introduction of kernel technology, SVMs have aided in the advancement of classification problems as machine learning has progressed. The main idea was to make problems easier to handle by mapping them from a low-dimensional space to a high-dimensional space, which was initially challenging to solve [12]. The secret to this approach is that feature mapping may make the initially linearly inseparable data linearly separable in high-dimensional space. The number of parameters could, however, rise noticeably as a result. Additionally, as the dimensions of the data rise, so does the computational complexity.

Researchers have started using CNN for heart sound feature extraction as deep learning has grown in popularity. A deep learning model that extracts special characteristics in the time axis was built by Christian Thomae et al. [13]. This network uses an attention strategy to weight the time step size and includes convolutional and recurrent layers. Lastly, they obtained a validation score of up to 89% using dense multi-layer perceptrons as classifiers. However, complicated heart sound patterns cannot be properly represented by a single spectral characteristic. This problem was identified by Devjyoti Chakraborty et al. [14], who suggested a 2D CNN model that takes the heart wave spectrum as input. They extracted more feature information by turning cardiac sound waves into spectrograms, which allowed them to record the spread and variations of the sounds at various frequencies. Even though this approach somewhat enhances feature extraction capabilities, temporal information still needs consideration. By creating a temporal mask, Li et al. [15] produced pseudo-based TP and QRS complex data. They then created an enlarged residual CNN structure to increase the atrial fibrillation diagnosis achievement in wearable ECG recordings to 0.843. In their 2016 Physionet Challenge database, Riccio et al. [16] achieved a correction accuracy of 0.85 using an end-to-end CNN model and the partition iteration function technique using fractal methods for images.

A general review of the literature reveals numerous machine learning-based studies on the detection of cardiovascular diseases from PCG signals. The vast majority of these recent studies involve deep learning models. The purpose of using these models is to improve classification performance. The 2016 PhysioNet/CinC Challenge dataset, which contains PCG signals, was chosen for the present study. This dataset is a popular dataset with over 19,000 citations. The methodological contributions of the proposed approach are as follows:

- By converting raw PCG signals into spectrogram images, the data’s representativeness is increased in the frequency and time domains. Furthermore, it becomes possible to work with two-dimensional deep learning models.
- Strong deep features are extracted from a specialized MLP-Mixer structure, where attention and residual structures can work in parallel.
- A hybrid feature selection algorithm combining the strengths of NCA and ReliefF structures is used, thus improving classification performance. The added value of the NCA- and ReliefF-Based Matching Indexing (NRBMI) lies not in introducing an entirely new algorithm but in its intersection-based validation strategy. By requiring features to be simultaneously important according to NCA and ReliefF, NRBMI minimizes noise and irrelevant variables, resulting in improved generalization and more balanced classification performance.

Although spectrogram transformation, attention mechanisms, residual connections, MLP-Mixer structures, feature selection methods, and SVM classifiers have each been employed individually in prior studies, the novelty of this work lies in the systematic integration of these components into a single framework. By combining these complementary elements, our approach leverages both time–frequency representations and advanced deep feature extraction strategies, leading to a more robust and balanced classification performance compared to existing methods.

## 2. Materials and Methods

**Figure 1:**
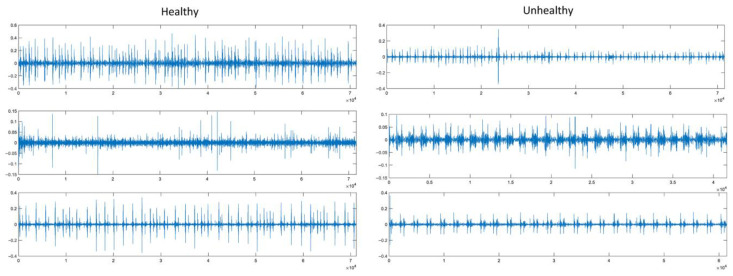
PCG signal samples from the 2016 PhysioNet/CinC Challenge dataset.

Encouraging the creation of algorithms to categorize heart sound recordings taken from various clinical or nonclinical (such as in-home visits) settings is the goal of the 2016 PhysioNet/CinC Challenge [17]. The objective is to determine whether the subject of a single, brief recording (10–60 s) from a single precordial region needs to be sent for a professional diagnosis. Atrial and ventricular contractions are caused by electrical activity that is first generated by the heart during the cardiac cycle. Blood is then forced throughout the body and between the heart’s chambers as a result. The entire cardiac structure vibrates as a result of the operations of the heart valves, which are linked to blood accelerations and decelerations and produce heart sounds and murmurs. The chest wall can produce these vibrations, and listening for particular heart sounds can reveal information about the condition of the heart. A recording of heart sounds is represented graphically by a phonocardiogram (PCG). A total of 3541 original PCG recordings in *.wav format are included in the collection. Of these PCG signals, 2725 were labeled as healthy and 816 as unhealthy by expert physicians. The majority of the data in the validation dataset comes from the training set. Since the official test dataset is not made available to the public, it is not included here. At 2000 Hz, the PCG is resampled. Three examples of healthy and unhealthy classes from PCG signals in the dataset are given in Figure 1.

**Figure 2:**
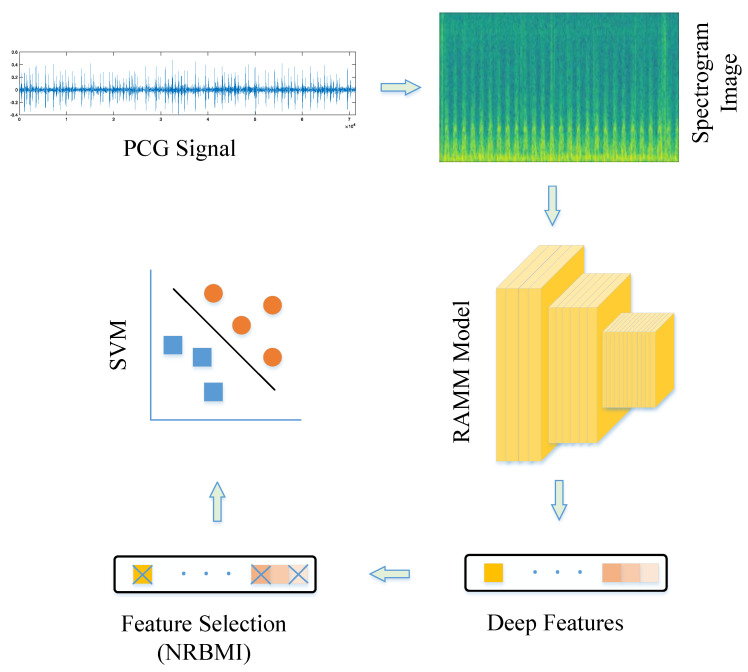
The representation of the proposed strategy.

**Figure 3:**
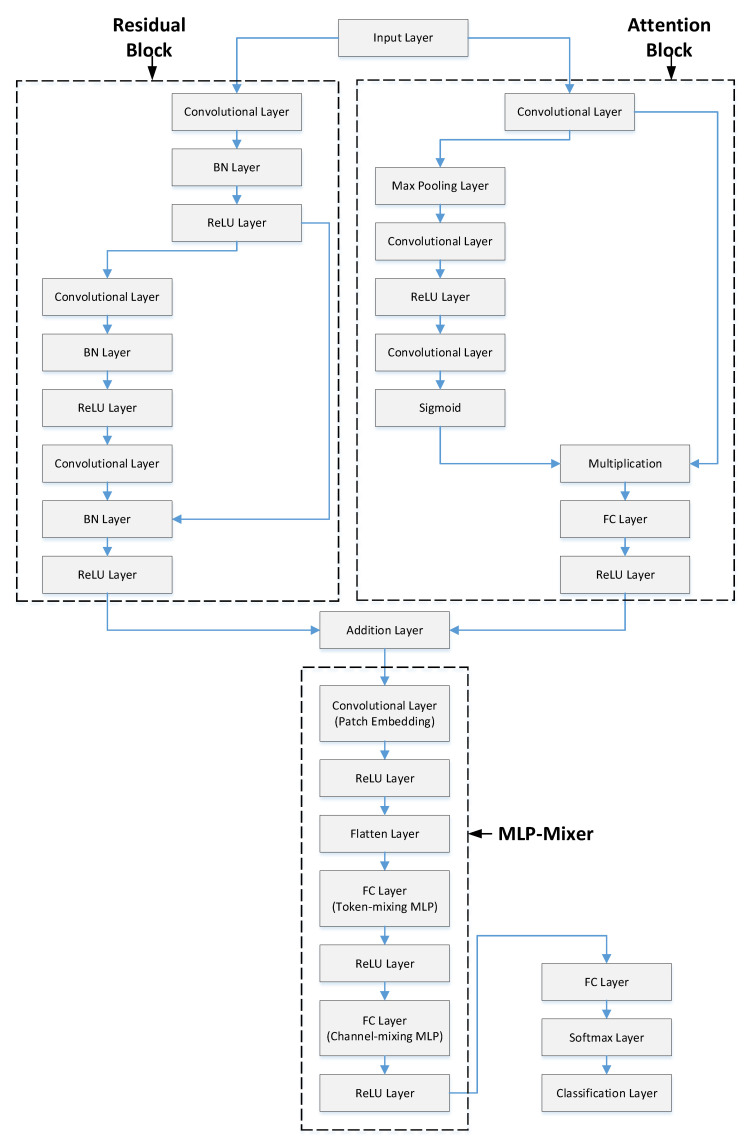
Layer structure of the RAMM model.

The proposed strategy includes five stages. Figure 2 shows a representative illustration of this methodology. In the first stage, PCG signals from the PhysioNet/CinC Challenge dataset were read and converted into spectrogram images. Spectrograms provide useful information in both frequency and time domains, increasing data representativeness. Converting signals to 2D made them suitable for 2D CNN models and removed the need for resampling to a fixed size. In the second stage, training and validation were performed using the RAMM model. A representative representation of the RAMM model is shown in Figure 3. In the RAMM model, attention and residual structures are connected in parallel. This setup allows both advantages to be used together. Their outputs are then transferred to the MLP-Mixer structure. In the third step, features were extracted from the final ReLU layer of the RAMM network. Instead of using a softmax classifier, these outputs were tested with the SVM algorithm, which is a more powerful classifier. In the fourth stage, instead of using the entire feature set, feature selection was performed using the NCA- and ReliefF-Based Matching Indexing (NRBMI) algorithm, which combines the strengths of the NCA algorithm and the ReliefF algorithm. This new hybrid algorithm reduced the size of the feature set and improved classification performance. In the fifth stage, the SVM algorithm was used. This is a popular method in machine learning and performs well in most classification tasks.

A spectrogram is a two-dimensional representation of a signal’s frequency content, which varies over time. Time information is displayed on the horizontal axis, and frequency information is displayed on the vertical axis [18]. The color or intensity in spectrogram images represents the amplitude of the frequency components. The Short-Time Fourier Transform (STFT) method is typically used to obtain a spectrogram [19]. The signal is divided into small time windows, and the Fourier transform is performed for each window.

Heart sounds such as S1, S2, and murmurs are short-lived signals with characteristic frequency components. While classical FFTs cannot show their time distribution, spectrograms can. This allows the following:

- Time and frequency resolution can be achieved simultaneously.
- Differences in duration, interval, and intensity of heart sounds can be visualized.
- Pathological conditions such as murmurs can be distinguished by their frequency patterns.

Deep learning models can process these 2D images for classification or anomaly detection.

**Figure 4:**
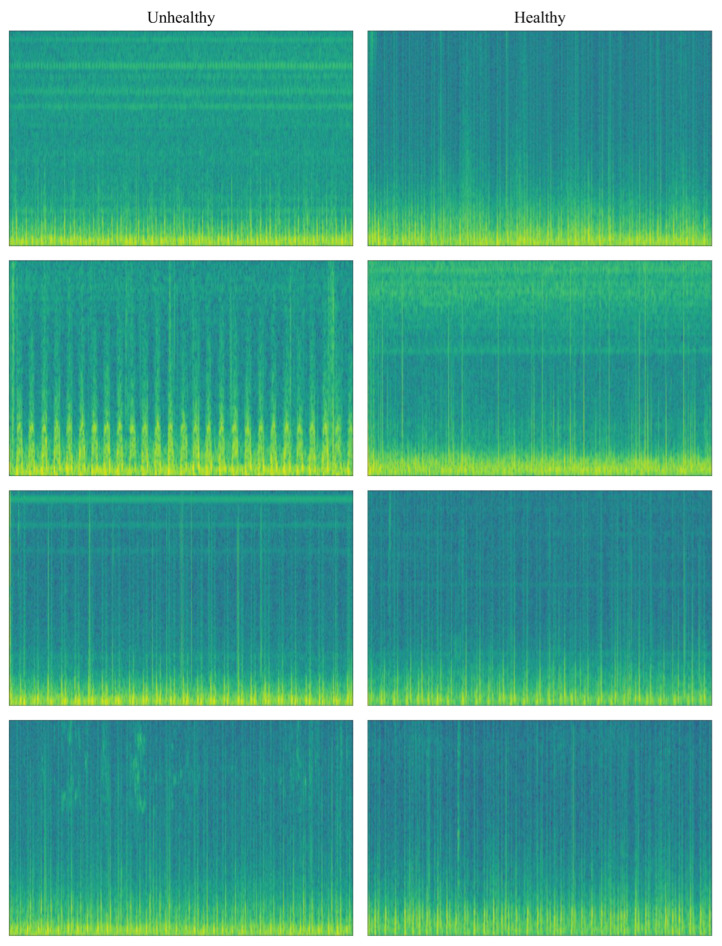
Spectrogram transformations illustrating differences between healthy and unhealthy PCG signals.

In Figure 3, spectrogram image transformations of PCG signals are given according to class separation. Looking at Figure 4 and the color tones, it can be seen that there is a difference in the color patterns between the classes.

Residual structures were first introduced with the ResNet architecture [20]. Their primary purpose is to address the vanishing gradient and gradient performance problems that arise during training in deep neural networks. Residual structures offer many advantages. Residual connections allow the input to be passed directly to the next layer. This results in more stable gradient distributions, and even in deep networks, learning accuracy remains stable. Thanks to residual structures, models with 50, 101, and even 1000 layers can be trained. As depth increases, the number of complex relationships that can be learned increases [21]. Passing input data directly to subsequent layers prevents the model from having to memorize as much, which improves generalization. Deeper models with residual connections train faster and more stably than traditional models of the same depth.

The model may concentrate on significant portions of the input data thanks to the attention mechanism. This is an essential component of contemporary AI models like Vision Transformer (ViT), CNN, BERT, and Transformer. Deep learning models benefit greatly from the attention mechanism. Each input’s contribution is dynamically weighted in attention structures. For instance, the model focuses more on a keyword in a sentence or a particular object in a picture. Relationships between data can be efficiently modeled by the attention mechanism, particularly when dealing with sequential data. This offers a major benefit over LSTMs and RNNs. Attention structures, as opposed to RNNs, facilitate parallel processing, which enables quicker GPU training and inference. The interpretability of model decisions is improved by visualizing the attention weights, which makes it easier to see what the model is concentrating on.

MLP-Mixer is a notable visual classification model proposed by Google in 2021. Unlike conventional CNN and Transformer structures, it uses only multi-layer perceptrons (MLPs). In this respect, it offers an innovative approach [22]. MLP-Mixer divides an image into fixed-size patches and then performs learning on these patches using only MLP layers. It consists of two main components: Patch (token) mixing MLP, which learns relationships between different spatial regions (patches), and Channel mixing MLP, which learns relationships within the channel (feature) dimension of the same patch. MLP-Mixer models offer many advantages. The most significant advantage is that they provide high performance without requiring very large and complex structures [23]. This allows good performance to be achieved with fewer hardware requirements. Because matrix multiplication is at the forefront, they are well suited to parallelization on GPUs and TPUs. Token mixing learns dependencies between patches, while Channel mixing processes the internal structure of each patch. This two-dimensional decoupled learning is like an abstract equivalent of the operation performed by kernel filters in CNNs.

The proposed RAMM model combines these three important structures into a single model, making significant contributions to improving classification performance.

- Parallel and simultaneous training extracts both residual structural information and attention contextual information on the same input. This provides a richer representation.
- Connecting the residual and attention outputs to the MLP-Mixer allows mixing of this rich feature set at both the spatial and intra-channel levels.
- Because the features from the residual and attention models are activated by the ReLU layer, the model converges more stably and quickly.

### NCA- and ReliefF-Based Matching Indexing (NRBMI) Algorithm

NCA is an algorithm developed by Jacob Goldberger and Geoffrey Hinton in 2005 [24]. Its primary goal is to find a linear transformation that re-represents examples in the data space to improve classification accuracy. This transformation results in examples from similar classes being closer together and examples from different classes being further apart [25]. The main goal of NCA is to transform the data and optimize the neighborhood relationships with the k-nearest neighbor (KNN) classifier system. The 1-NN classifier is mostly used. If the set containing the training data X is defined as the set containing the class labels Y, the aim is to find the ideal linear transformation matrix $A\in R^{m\times d}$. In this matrix, *m* is the feature dimension and *d* is the output dimension. In NCA, the probability of choosing another point *j* for each point *i* is given in Equation (1). This equation works according to the softmax principle.

(1) $$p_{ij}=\frac{e^{{-\left\|{Ax}_{i}-{Ax}_{j}\right\|}^{2}}}{\sum_{k\neq i}e^{{-\left\|{Ax}_{i}-{Ax}_{k}\right\|}^{2}}}$$

Here, *k* represents the size of all indexes except the *i*th sample. The total probability of confirmation is given in Equation (2).

(2) $$f\left(A\right)=\sum_{i}\sum_{j\;\in \;same\;class}p_{ij}$$

Using feature weights, the ReliefF algorithm generates effective feature predictions. The convex optimization problem is solved to get the feature weights [26]. To find the nearest k value, the weights of all features are first set to 0. At each stage, the data is then randomly selected from the data set [27]. The closest data for each specific class is then retrieved [28]. The feature weights are then updated using this data. Ultimately, a new data set is created once an inadequate feature for the specified criterion has been eliminated from the original data set. The ReliefF method is formulated as follows in Equation (3).

(3) $$W{(x}^{a})=W{(x}^{a})-\frac{\sum_{j=1}^{k}diff(A,R_{i},H_{j})}{mxk}+\sum_{c\neq class\left(R_{i}\right)}\left[\frac{P\left(C\right)}{1-P(class(R_{i}))}x\sum_{j=1}^{k}\frac{diff(A,R_{i,M_{j}})}{mxk}\right]$$

where ${(x}^{a})$ represents the ath feature, *A* represents the feature set, $R_{i}$ and $H_{j}$ represent samples of the feature data, and *k* represents the manual parameter.

In the developed NCA- and ReliefF-Based Matching Indexing (NRBMI) algorithm, feature indices are first ranked according to the weights calculated by the ReliefF and NCA techniques. The index values of the feature data with the optimum “x” weights provided from the ReliefF and NCA methodologies are compared. The features with matching index values constitute the output of the NRBMI algorithm (see Algorithm 1).

The strength of the NRBMI algorithm lies in its dual-perspective validation of feature importance. By combining NCA’s classification-driven feature ranking with ReliefF’s distance-based relevance assessment, NRBMI selects only the features deemed important by both methods. This intersection-based strategy reduces noise, prevents irrelevant feature inclusion, improves generalization, and yields a more compact yet highly informative feature subset.
**Algorithm 1.** Pseudocode of the NRBMI Algorithm**Input:**  Dataset D with features F = {f1, f2, …, fn}
  Labels Y
  Parameter x //number of top features to select from each method
**Output:**  Selected feature set S
**Steps:**1. Compute feature weights using NCA:    W_NCA = NCA_Weights(D, Y)
    //W_NCA is an array of length n with importance scores
2. Sort features by W_NCA in descending order:    Sorted_NCA = sort_indices_by_weight(W_NCA, descending=True)
3. Select top x feature indices from NCA results:    Top_NCA_Indices = first_x_indices(Sorted_NCA, x)
4. Compute feature weights using ReliefF:    W_ReliefF = ReliefF_Weights(D, Y)
    //W_ReliefF is an array of length n with importance scores
5. Sort features by W_ReliefF in descending order:    Sorted_ReliefF = sort_indices_by_weight(W_ReliefF, descending=True)
6. Select top x feature indices from ReliefF results:    Top_ReliefF_Indices = first_x_indices(Sorted_ReliefF, x)
7. Find matching feature indices between NCA and ReliefF selections:     Matching_Indices = intersection(Top_NCA_Indices, Top_ReliefF_Indices)
8. Select features corresponding to Matching_Indices from D:    S = { f_i | i ∈ Matching_Indices }9. Return S

## 3. Results

All coding was performed in MATLAB 2024a on a Windows 11 PC with an Intel i9 processor, 12 GB RAM, and an RTX 3080ti GPU. Spectrogram images were generated to train the RAMM model. Hamming was used for windowing to create the spectrogram images, and the size was set to 400. The overlap value and FFT size were set to 50. Normalization was performed only in the frequency domain and applied as pi./[samples]. Furthermore, all images were resized to 200 × 200. The total number of learnable parameters of the RAMM model is 582k, and the parameter memory size of the model is 2.13 MB. This indicates that the RAMM model is approximately seven times lighter than MobileNetV2, one of the lightest CNN models.

**Figure 5:**
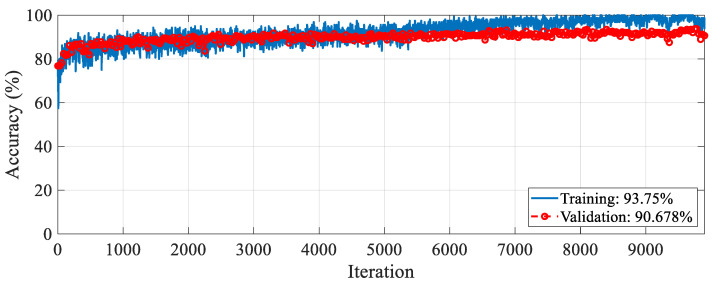
Accuracy graph of the RAMM model with spectrogram images during the training process.

**Figure 6:**
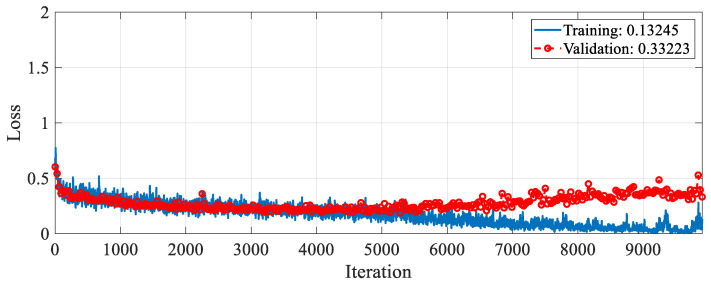
Loss graph of the RAMM model with spectrogram images during the training process.

The proposed RAMM model was trained for feature extraction. For training settings, the optimizer, epoch, and learning rate were set to SGDM, 50, and 0.001, respectively. The validation method employed was 10-fold cross-validation, and the loss function was cross-entropy. The accuracy and loss change graphs of the RAMM model during the 9900 iteration training process are given in Figure 5 and Figure 6, respectively. At the end of training, accuracy reached 93.75%, and validation accuracy reached 90.67% (Figure 5). Training loss was 0.02, while validation loss was 0.3 (Figure 6).

**Figure 7:**
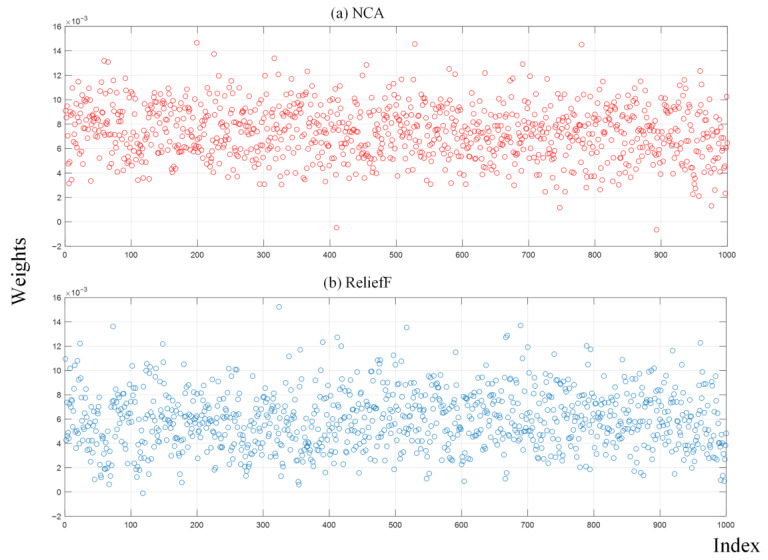
Feature weights with NCA and ReliefF algorithms.

**Figure 8:**
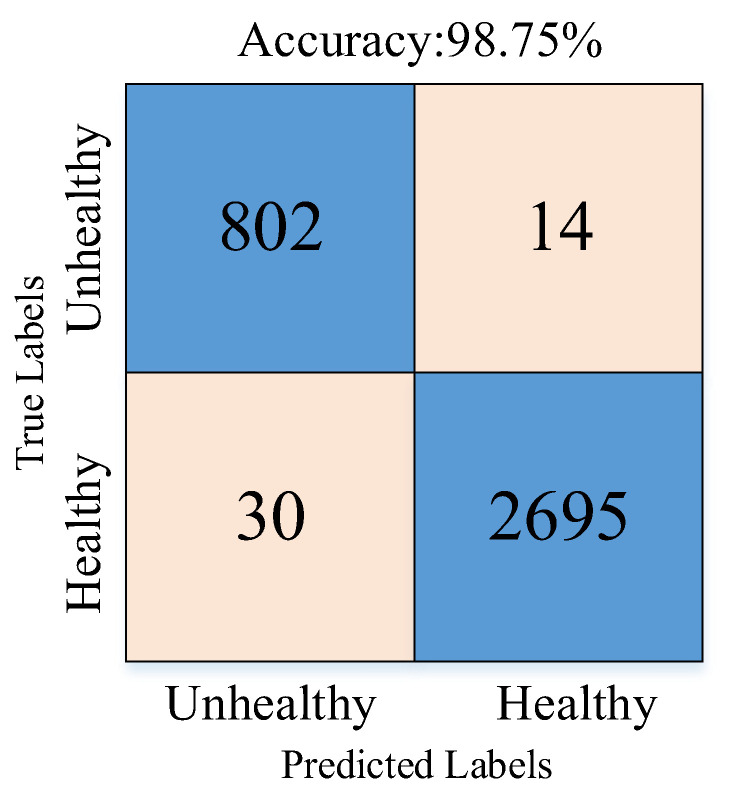
Confusion matrix depicting the performance of the proposed RAMM model with the NRBMI algorithm.

Next, 1000 features were extracted from the last ReLU layer of the RAMM model. Their importance weights were calculated with both the NCA and ReliefF algorithms, and the NRBMI algorithm was applied for feature selection. The importance weights of these extracted features were calculated by both the NCA and ReliefF algorithms. The results of the calculated importance weights are given in Figure 7. In the NRBMI algorithm, the top 300 features from NCA (Figure 7a) were compared with the top 300 from ReliefF (Figure 7b). A total of 128 features matched, and these were passed to the SVM classifier. The best results in the SVM algorithm were obtained with the polynomial kernel. The classification results obtained with the SVM algorithm are given as a confusion matrix in Figure 8. Figure 8 shows that the proposed approach correctly classified 2695 healthy and 802 unhealthy samples. It misclassified 30 healthy and 14 unhealthy samples.

Table 1 presents the sensitivity, specificity, precision, and F-score metrics calculated using values from the confusion matrix. These metrics were used because the number of samples was unbalanced across classes. Table 1 shows that all metrics exceeded 95%. Sensitivity and specificity were balanced at about 98%, while precision and F-score differed by 3% and 2%, respectively.

In the study, no resampling techniques were applied to address the class imbalance (2725 healthy vs. 816 unhealthy). Instead, 10-fold cross-validation was employed, and additional performance metrics such as sensitivity, specificity, precision, and F1-score were reported to mitigate the impact of imbalance. Furthermore, the NRBMI feature selection algorithm contributed to balanced performance across both classes by prioritizing highly discriminative features. Also, the confusion matrix results of many experiments are given to clearly show the imbalance between class numbers.

To increase the reliability of the proposed approach, the 10-fold cross-validation technique was run 40 times. The validation accuracy results are given in Table 2. According to these results, the average accuracy value was 98.2%, while the standard deviation value was 0.45.

## 4. Discussion

**Figure 9:**
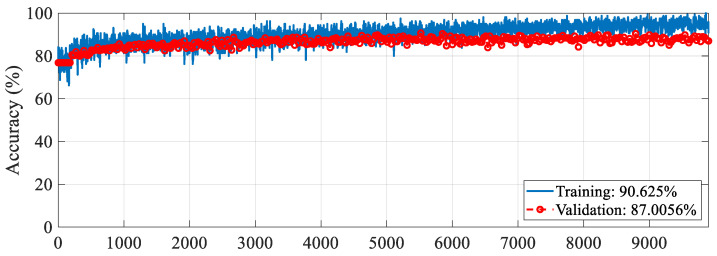
Accuracy graph of the RAMM model with raw data during the training process.

**Figure 10:**
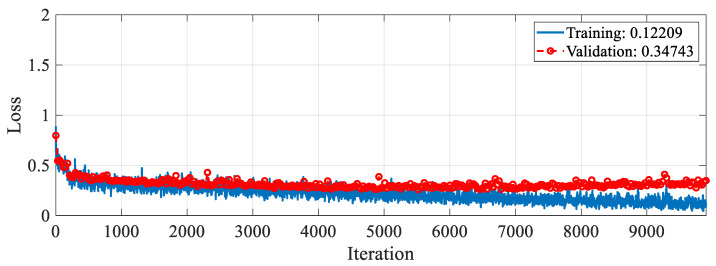
Loss graph of the RAMM model with raw data during the training process.

This section presents ablation studies to show the effectiveness of the proposed approach and compares it with other methods using the same dataset. The first ablation study was conducted to demonstrate the effectiveness of spectrogram images. In this study, training was conducted on the RAMM model using the training options. Because the raw data is a one-dimensional signal, the layers in the RAMM model were transformed to operate in one dimension. Under these conditions, the training and validation accuracy changes obtained during the 9900 training iterations are presented in Figure 9, while the training and validation loss changes are presented in Figure 10. At the end of the training, the training accuracy was 90.5%, while the validation accuracy was 87.4%. These results show that using spectrogram images improved validation accuracy by about 3.6%.

**Figure 11:**
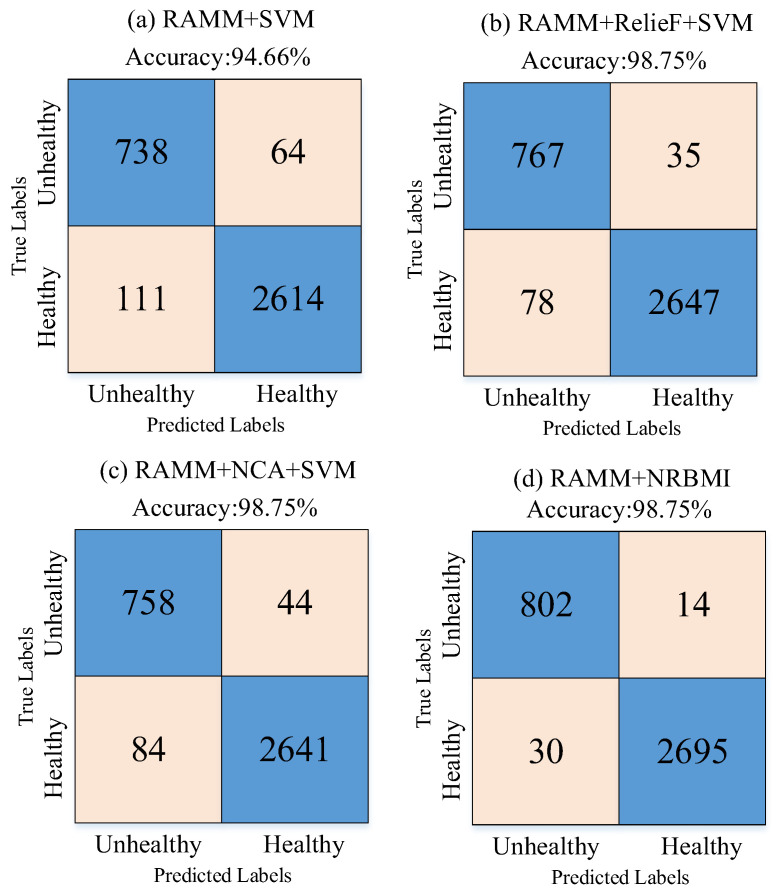
Confusion matrix results of ablation studies for the NRBMI algorithm: (**a**) RAMM model + no feature selection + SVM classifier, (**b**) RAMM model + ReliefF feature selection + SVM classifier, (**c**) RAMM model + NCA feature selection + SVM classifier, (**d**) RAMM model + NRBMI feature selection + SVM classifier.

The experimental results obtained in Figure 11 and Table 2 are given to demonstrate the effectiveness of the NRBMI algorithm. Figure 11 shows that when RAMM features are fed directly to the SVM classifier (Figure 11a), accuracy stays at 94.66%. The errors are relatively high, especially in the healthy class, with 111 misclassifications. Using ReliefF for feature selection (Figure 11b) reduced errors and increased accuracy to 98.75%. The NCA method (Figure 11c) achieved the same accuracy, with 84 misclassifications. With the proposed NRBMI algorithm (Figure 11d), misclassifications dropped to the lowest levels (30 and 14). It achieved a more balanced success across classes while keeping accuracy at 98.75%.

Table 2 confirms these findings. In the RAMM + SVM model, sensitivity for the “Healthy” class was 95.93%, and specificity was 92.01%. For the “Unhealthy” class, sensitivity and specificity were 92.01% and 86.94%. While ReliefF- and NCA-based selections significantly improved these values, the highest and most balanced performance was achieved with the NRBMI algorithm. For example, the RAMM + NRBMI approach achieved results above 98% in sensitivity, specificity, precision, and F1-score values for both the “Healthy” and “Unhealthy” classes. In particular, the sensitivity (98.28%) and precision (96.39%) values for the “Unhealthy” class demonstrate higher and more balanced classification success compared to the other methods.

Together, Figure 11 and Table 3 show that the NRBMI algorithm improves accuracy and keeps class balance, making classification more reliable. These findings reveal that NRBMI-based feature selection maximizes the discriminatory power of features obtained from the RAMM model and significantly improves the performance of the SVM classifier compared to other methods.

Table 4 compares our results with existing studies on the 2016 PhysioNet/CinC Challenge dataset. Accuracy is the main metric, but specificity, sensitivity, and F1-score are also included. Xiao et al. [29] tested two CNN models: one with Clique blocks and one with Dense blocks. Both achieved over 94% accuracy. Specificity was above 96%, but sensitivity and F1-score stayed below 90%. Qiao et al. [30] obtained HS vectors from PCG signals. These HS vectors were tested on the Time-Compressed and Frequency-Expanded TDNN (TCFE-TDNN) module. With this strategy, the accuracy, specificity, F1-score, and sensitivity values were 95.6%, 97.7%, 89.2%, and 87.6%, respectively. Xiao et al. [1] addressed the classification problem in two different scenarios. In the first scenario, features obtained from 1D and 2D CNN models were tested on DNN and KNN classifiers. In the second scenario, manual features were extracted using the MFCC algorithm, combined with features obtained from the 2D CNN model, and fed back to the same classifiers. Both strategies yielded very similar performance results, with classification accuracy exceeding 96%. Tian et al. [31] trained a Res2Net CNN model for heart disease detection. It reached 91% accuracy and 95% specificity, but sensitivity and F-score were below 80%. Xiao et al. [32] trained a 1D CNN model end-to-end and evaluated it on a dataset to detect heart disease. This model achieved 93% classification accuracy, 86% specificity, 81% F1-score, and 95% sensitivity. Alkhodari et al. [33] used a 1D CNN model to extract deep features from PCG signals. These extracted features were then fed into an RNN model for classification. This strategy resulted in lower accuracy than other models, achieving 88.2%. Duan et al. [34] employed three different strategies to classify PCG signals. They used three different techniques for this purpose. The first technique created a block structure called a para block that could train a structure containing convolutional, ReLU, and pooling layers in four different branches in parallel. The second technique created attention block structures. The third technique used the matrix product state (MPS) algorithm, a competition technique for high-dimensional data. The first model used only para blocks. The second model used the para block and MPS algorithms. The third model used a combination of para block, attention block, and MPS algorithms. The best results were obtained with the third model. The proposed approach outperformed other approaches using the same dataset in terms of accuracy, sensitivity, and F1-score. It fell short of the best performance by only 0.8% in terms of specificity. It outperformed the best performance by 2.4% in terms of accuracy. However, due to differences in training parameters and evaluation protocols, it would be inappropriate to claim absolute superiority over other models with similar performance. Nevertheless, the proposed approach consistently exceeds 98% accuracy across four performance metrics on a widely used dataset, indicating it can be a promising and reliable candidate for decision support systems; nonetheless, further validation on external, real-world data is needed. In real-world applications, the following strategies will be pursued to ensure clinical translation and robustness of the proposed approach: (1) incorporating realistic noise and device-response augmentations at multiple SNR levels to test tolerance against environmental interference, (2) evaluating performance after downsampling and audio compression to mimic wearable and smartphone recording pipelines, and (3) employing domain-adaptation techniques by fine-tuning the model on a small set of recordings from wearable devices. Furthermore, we will include calibration metrics (Brier score, reliability diagrams) and uncertainty estimation methods (e.g., deep ensembles) to monitor model confidence under noisy or device-specific conditions.

Although the proposed method offers high accuracy, this study has some limitations. First, the 2016 PhysioNet/CinC Challenge dataset used was collected under controlled conditions, and performance may suffer from noisy, incomplete, or low-quality data in real-world scenarios. In the future, environmental noise, device variations, and patient motion—especially in heart sounds recorded at home or in busy clinical settings—may pose significant challenges to the model. Furthermore, the high computational requirements of deep learning-based models may limit their application on portable devices or low-cost hardware. In this context, the development of lighter models or model compression/optimization techniques is crucial. Furthermore, issues such as patient privacy, data security, and the ethical responsibility of AI-based decisions should be considered in the widespread use of clinical decision support systems. Future studies should focus on developing new methods that address these limitations and validate them with multicenter, real-world patient data. For the proposed approach to be effectively used in clinical applications, digital stethoscopes capable of transmitting recordings via radio waves are required. Data collected from these stethoscopes can be transmitted to AI servers. After evaluation on these servers, it can be sent to mobile and web applications via cloud-based systems. Device sensitivity and patient diversity during data acquisition may pose challenges to the general validity of the proposed model. To prevent this, training and testing with new datasets must be conducted with clinical validation.

Experimental results of the proposed approach demonstrate that the main contribution of this study is not the introduction of entirely new modules but rather the effective integration of spectrogram-based representations, residual and attention mechanisms, MLP-Mixer structures, and a hybrid feature selection strategy within a unified pipeline. This integration allows the strengths of each component to complement one another, resulting in improved generalization and balanced performance across classes. Thus, the novelty of our approach lies in the synergy achieved through the combined use of these well-established techniques.

## 5. Conclusions

Heart sounds are an important data source for diagnosing heart conditions. However, expert interpretation is needed for accurate interpretation of this data. Artificial intelligence-powered applications that automate these interpretations are needed for early diagnosis. Therefore, this study was conducted on the popular 2016 PhysioNet/CinC Challenge dataset. The proposed lightweight RAMM model extracted highly discriminatory features. Highly representative features were selected using the newly developed NRBMI algorithm. The popular and powerful SVM algorithm was used for classification. The proposed approach achieved over 98% success in all metrics: accuracy, sensitivity, specificity, precision, and F1-score. Detailed experimental studies were conducted to demonstrate the effectiveness of the techniques used in the proposed approach. Performance comparisons were also conducted with other studies using the PhysioNet/CinC Challenge dataset. While the proposed model achieved high accuracy within the PhysioNet/CinC dataset, this performance is limited to 10-fold cross-validation. Therefore, further validation on independent datasets and prospective clinical studies is necessary before the model can be translated into real-world clinical practice. As a result, a highly reliable decision support system has been developed. Future tests with real-world data are planned after obtaining the necessary ethics committee approvals. This software application may also be adapted to wearable technologies in the future.

## Figures and Tables

**Table 1:** Performance metric values of the proposed approach.

Class	Sensitivity (%)	Specificity (%)	Precision (%)	F1-Score (%)
Healthy	98.90	98.28	99.48	99.19
Unhealthy	98.28	98.90	96.39	97.28

**Table 2:** Accuracy scores of the proposed approach for 10-fold cross-validation.

Number of Cross—Validation →	1–10	11–20	21–30	31–40
Results **→**	98.59%	99.44%	98.36%	99.52%
	98.61%	98.22%	98.22%	99.21%
	98.33%	99.50%	98.39%	98.11%
	98.66%	99.04%	99.41%	99.24%
	98.45%	99.52%	98.93%	98.03%
	98.59%	97.89%	98.70%	98.70%
	98.61%	98.81%	99.41%	98.53%
	98.75%	98.11%	98.17%	98.19%
	98.89%	98.75%	98.47%	99.01%
	99.38%	99.21%	98.53%	99.18%

**Table 3:** Performance metric results of ablation studies for the NRBMI algorithm.

Model	Class	Sensitivity (%)	Specificity (%)	Precision (%)	F1-Score (%)
RAMM + SVM	Healthy	95.93	92.01	97.62	96.77
	Unhealthy	92.01	95.93	86.94	89.42
RAMM + ReliefF + SVM	Healthy	97.13	95.65	98.70	97.91
	Unhealthy	95.65	97.13	90.75	93.12
RAMM + NCA + SVM	Healthy	96.91	94.50	98.36	97.63
	Unhealthy	94.50	96.91	90.01	92.19
RAMM + NRBMI	Healthy	98.90	98.28	99.48	99.19
	Unhealthy	98.28	98.90	96.39	97.28

**Table 4:** Evaluation against existing techniques with the 2016 PhysioNet/CinC Challenge dataset.

Methods	Accuracy (%)	Specificity (%)	F1-Score (%)	Sensitivity (%)
CliqueCNN [29]	94.01	96.81	85.13	83.21
DenseCNN [29]	94.21	96.62	85.8	84.92
HS-based Vectors [30]	95.62	97.72	89.2	87.61
1D CNN + 2D CNN [1]	96.32	97.92	91.11	90.61
1D hand-crafted feature + 2D CNN [1]	96.22	97.94	90.62	89.72
Res2Net-CNN [31]	91.03	95.01	77.52	74.51
1D CNN [32]	93.01	86.01	91.02	95.02
1D CNN and RNN [33]	88.22	88.92	74.91	85.92
ParaCNN [34]	94.61	94.22	86.33	85.13
ParaMPS [34]	96.10	98.70	88.71	86.03
Params [34]	96.40	99.10	89.30	86.50
Ours (RAMM + NRBMI + SVM)	98.80	98.30	99.20	98.90

## Data Availability

The original data presented in the study are openly available in https://www.kaggle.com/datasets/swapnilpanda/heart-sound-database (accessed on 17 September 2025).
